# Lithography-Free Water Stable Conductive Polymer Nanowires

**DOI:** 10.1021/acs.nanolett.4c05016

**Published:** 2025-02-13

**Authors:** Damien Hughes, Abdelrazek H. Mousa, Chiara Musumeci, Malte Larsson, Muhammad Anwar Shameem, Umut Aydemir, Ludwig Schmiderer, Jonas Larsson, Magnus Berggren, Fredrik Ek, Roger Olsson, Martin Hjort

**Affiliations:** † Chemical Biology & Therapeutics, Department of Experimental Medical Science, Lund University, SE-221 84, Lund, Sweden; ‡ Department of Chemistry and Molecular Biology, University of Gothenburg, SE-405 30, Gothenburg, Sweden; § Laboratory of Organic Electronics, Department of Science and Technology, Linköping University, SE-60174, Norrkoping, Sweden; ∥ Division of Molecular Medicine and Gene Therapy, Department of Laboratory Medicine and Lund Stem Cell Center, Lund University, SE-221 00, Lund, Sweden; ⊥ Chemical Biology Consortium Sweden (CBCS), Karolinska Institute, S-171 21, Stockholm, Sweden

**Keywords:** nanowires, PEDOT-S, bioelectronics, cellular interfacing, conductive polymer, algae

## Abstract

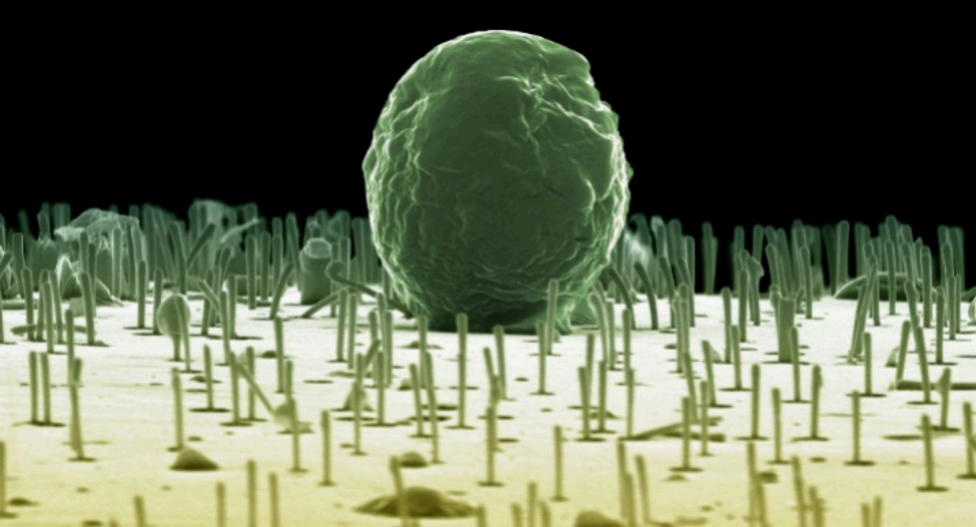

Free-standing nanowires can gain intracellular access without causing stress or apoptosis. Current approaches to generate nanowires focus on lithographic patterning and inorganic materials (Si, GaAs, Al_2_O_3_, etc.) while organic materials are less explored. Use of organic conductive polymers allows for the creation of soft mixed ion–electron conducting nanowires. Processing conductive polymers into nanowires is challenging due to the harsh chemicals and processing conditions used. Here, we demonstrate a lithography-free and scalable method to generate all-organic, water-stable nanowires composed of conductive polymers. A nanoporous membrane is filled with conductive polymer in solution, followed by a cross-linking step to make the polymer water stable. The surface of the membrane is anisotropically etched using a reactive ion etcher to reveal the polymer inside the pores, which extends from the membrane as nanowires. We interface the nanowires with model algal cells and human primary hematopoietic stem and progenitor cells.

Free-standing, axially elongated nanostructures, such as nanowires (NWs), are ideal for applications within solar cells,^1^ light-emitting diodes,^2^ and electronics.^3^ Lately, such nanostructures have been interfaced with living cells, making it possible to measure cellular traction forces,^4^ deliver biomolecules into or out of living cells without toxic effects,^5,6^ and for artificial photosynthesis.^7^ Due to their comparable ease of processability, these applications have so far been centered on nanostructures based on semiconductors,^8,9^ metals,^10,11^ or metal-oxides.^12,13^ A biocompatible carbon-based alternative is lacking.

Conductive polymers (CPs) are a class of materials being pursued for applications within bioelectronics due to their ability for mixed ion–electron conductivity^14,15^ and their soft, self-healable, flexible nature which allows for seamless interfacing with tissue and cell cultures.^4^ To comply with future needs and to make use of the very high surface-to-volume ratio provided by nanostructure geometry, miniaturization is needed while still being able to pattern large areas. Conventional photolithography and electron lithography are poorly suited for this task due to harsh etching conditions and a lack of possibility to clean the CP from photoresist and other contaminants.^16^

Here, we present a lithography-free method to make a bespoke CP NW platform that can be used to interface with living cells. The NWs are formed by poly(3,4-ethylenedioxythiophene)butoxy-1-sulfonate (PEDOT-S)^17^ templated in track-etched (TE) membranes and made water-stable through introduction of cations or by electrofunctionalization with small thiophene trimers.^18^ The methods allow full control over the geometry and chemistry of the NWs by choosing different templates, processing parameters, and incorporation of trimers with PEDOT-S. The NW dimensions and densities are ideal for interfacing with cells^19,20^ and even potential intracellular access.

The NW processing starts with a commercially available TE polyimide (PI) or polycarbonate (PC) membrane (It4IP) serving as a template, Figure 1A. The TE membrane is coated with CP followed by a gas-phase etch step, which selectively removes the template membrane, leaving the CP inside the pores to protrude as NWs. All dimensions of the NWs can be controlled by choosing a suitable starting membrane (density and diameter) and etch parameters (length).

**Figure 1:**
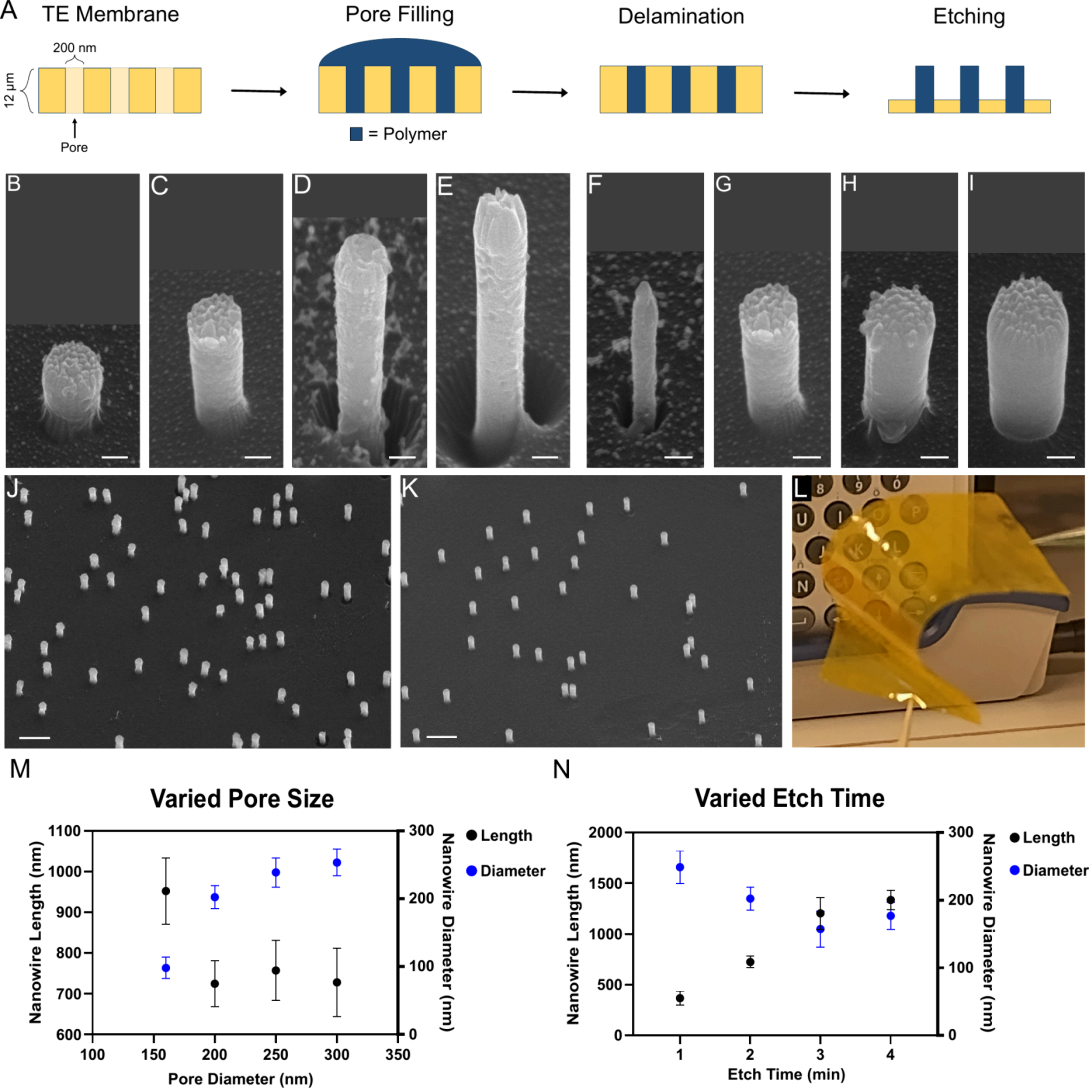
NW processing. (A) Schematic side view describing the processing. A track-etched (TE) membrane is filled with CP solution and then allowed to dry. After drying, excess CP is removed. The TE membrane is then dry-etched using oxygen-based inductively coupled plasma reactive ion etching (ICP-RIE) to reveal the NWs. Please note that the schematic is not to scale. (B–E) 30° tilted view SEM images of NWs of various lengths. Length can be controlled by modifying the etch time. The etching times used were 1 min (B), 2 min (C), 3 min (D), and 4 min (E). (F–I) 30° tilted view SEM images of NWs made to have different thicknesses due to varying pore diameter. The pore diameters used were 160 nm (F), 200 nm (G), 250 nm (H), and 300 nm (I). (J, K) 30° tilted SEM images showing different pore densities: 5.5 × 10^7^ cm^–2^ for panel J and 2 × 10^7^ cm^–2^ for panel K. (L) Photograph of processed NWs on polyimide membrane. The photograph showcases both flexibility and transparency of the material. (M, N) Graphs illustrating the effects of pore size (M) and etch time (N) on NW length and diameter. The dots on the graph represent the mean (14–33 NWs measured per dot), while the error bars represent the standard deviation. The scale bars are 100 nm in B–I and 1 μm in J and K.

Membranes suitable for cellular interfacing have pore densities in the 10^7^ cm^–2^ range and thicknesses of at least 10 μm to ensure the mechanical stability. The thickness of the membrane produces samples with high flexibility and optical transparency, making it possible to image through the membrane, Figure 1L, while the pore densities can determine how cells interface with the NWs. Higher pore densities result in denser NWs, and cells cultured on top of them will experience a bed-of-nails effect^21^ prohibiting a tight cell–NW interface, whereas lower densities risk some cells not being able to reach a NW. Since non-carbon NWs with a diameter in the range of 100–300 nm have been found to establish healthy connections to cells^6,22^ the same diameter range was chosen in this project.

Once the optimal TE membrane is determined, CPs in solution are added to the TE membrane to fill the pores. Pores become completely filled due to capillary forces. The physicochemical properties of the polymer solution are critical for efficient pore filling, guiding us to the use of self-doped PEDOT-S, which contains a covalently attached sulfonate group for charge injection. The PEDOT-S must be well-dispersed and highly water-soluble so as not to clog the pores or the pore openings. The most commonly used CP, poly(3,4-ethylenedioxythiophene):polystyrene-sulfate (PEDOT:PSS), did not provide sufficient pore filling. This is presumably due to the PSS which is comparably larger than PEDOT, and it can act to clog the pores. Further, even if PEDOT:PSS could fill the pores, there is a significant risk of phase separation, where the PSS contents at different heights of the pores would vary (and thus alter the conductive properties).

Previously, we have used a high concentration of PEDOT-S (20 mg/mL) to install substrate-free electrodes in animal models.^18,23^ While that concentration was possible to inject using very thin capillaries (30 μm), it could not efficiently fill the nanopores (100–300 nm) for the NW templating used here. To conserve reagents and achieve less aggregation in the CP solution, we used PEDOT-S at 1–10 mg/mL which resulted in NWs, Figures S1 and S10. Surface tension, as mapped by contact angle measurements, did not change when the PEDOT-S concentration was altered, Figure S11.

Once the polymer solution is applied, the sample is dried at room temperature before further processing. This leaves dried CP in the pores and on top of the sample. The dry top layer is mechanically delaminated using masking tape, Figure 1A, resulting in CP only being located within the pores. Lastly, the membrane is brought into an inductively coupled plasma reactive ion etcher (ICP-RIE), a dry-etcher commonly used within semiconductor processing. In ICP-RIE, a plasma is generated in a vacuum, and high-energy ions impinge on the sample to react with it. The CP inside the pores etch at a lower rate than the membrane, resulting in an isotropic etch where the NWs are seen to extend from the membrane. While this is the basic procedure for preparing NWs, modifications can be made to fit NWs to a specific need.

The length of the NWs can be tuned by using longer etch times or increasing the ICP-RIE power input. We found that 25 W RF power and 500 W ICP were high enough to have a useful etch rate while maintaining membrane integrity. It is important to minimize the thermal load on the sample to avoid wrinkling or degradation of the plastic membrane. This is especially important for polycarbonate (glass transition temperature, *T*_g_, 100–150 °C) but not as critical for polyimide (*T*_g_ > 300 °C). Figure 1B–E depicts representative NWs etched for 1–4 min showing a linear increase in length over time (320 nm/min). During longer etches, the NW diameter was observed to decrease (20 nm/min), allowing us to deduce a high etch selectivity of 16. Very long NWs reaching over 4 μm in length could also be processed without compromising NW structure (Figure S2).

NW diameter and density can be modified by choosing template membranes with different pore geometries. The nominal pore diameter, as defined by the vendor, was varied in between 160 and 300 nm (Figure 1F–I) and resulted in NWs ranging between 100 and 250 nm. The discrepancy stems from the vendors defining the average pore diameter in the membrane, whereas the NWs will only display the diameter at the top of the membrane, not taking the conicity of the pores into account. NWs made using the 160 nm pore membrane were slightly longer than ones made from the other membranes, indicating that porosity may affect the etch rate. Lastly, the density of NWs could be increased by using membranes with varying pore densities, Figure 1J,K, making them suitable to interface with cells of different sizes.

To interface with cells or biological fluids, the NWs must be water stable. While the NWs remain stable in organic solvents (e.g., acetone, isopropanol, ethanol, etc.), they readily dissolve in aqueous environments due to the high water solubility of the PEDOT-S. To combat this, we have devised two routes to increase the water stability: addition of cations or electrofunctionalization using thiophene trimers.

Metal cations increase the water stability of PEDOT-S through ionic cross-linking between the metal cation and the sulfonate group of PEDOT-S^17^ where higher valency cations exert more potent cross-linking. We added cations to the CP solution before placing it on the porous membrane. Trivalent cations, such as Fe^3+^, are the most efficient at stabilizing and can be added at mM concentrations without significantly affecting NW processing, Figure S1. The addition of trivalent cations is sufficient to increase water stability of NWs for up to 24 h when placed into PBS (Figure S4). Divalent cations such as Ca^2+^, Cu^2+^, and Fe^2+^ could also be used to generate NWs, Figure S3. However, care must be taken to balance the solubility, aggregation, and viscosity. Too high of an ion concentration combined with high PEDOT-S concentrations leads to polymer aggregating in the solution before addition to the TE membrane, leading to deficient pore filling (and thus no NWs), whereas too low ion concentration results in NWs which dissolve in water postprocessing (Figure S4).

Another route to increase water stability as well as to modulate the volume properties of the NWs is by incorporating small, nontoxic thiophene-based trimers.^18,23−25^ The trimers are composed of two ethylenedioxythiophenes (EDOTs) with a functionalized thiophene in the middle. The trimer used here, 4-(2-(2,5-bis(2,3-dihydrothieno[3,4-*b*][1,4]dioxin-5-yl)thiophene-3-yl)ethoxy)butane-1-sulfonate (ETE-S), is functionalized with a sulfonate group. Trimers such as ETE-S can be added to the CP solution and subsequently attached to PEDOT-S to increase NW stability. Incorporation can be done by applying an electrical bias (electrofunctionalization) or by applying an iron(III) catalyst (chemical functionalization).^18^ To electrofunctionalize ETE-S onto the PEDOT-S, an electrical bias is applied to the polymer while it is still wet and freshly added to the pores. For contacting, the TE membrane is gently placed onto a conductive tape serving as the anode and a AgCl dip-in electrode is used as the counter electrode. A bias of 0.9 V was applied between the electrodes throughout the electrofunctionalization. After the sample was dried overnight, ethanol was used to remove any conductive tape residues and excess polymer from the surface of the sample after delamination. NWs electrofunctionalized with ETE-S are shown in Figure 2K–M. While we present the incorporation of ETE-S into the NWs, more recent ETE derivatives harboring other chemical moieties (e.g., zwitterionic phosphatidylcholine or positively charged trimethylammonium^24^) can also be used to alter NW chemistry using the same methodology.

**Figure 2:**
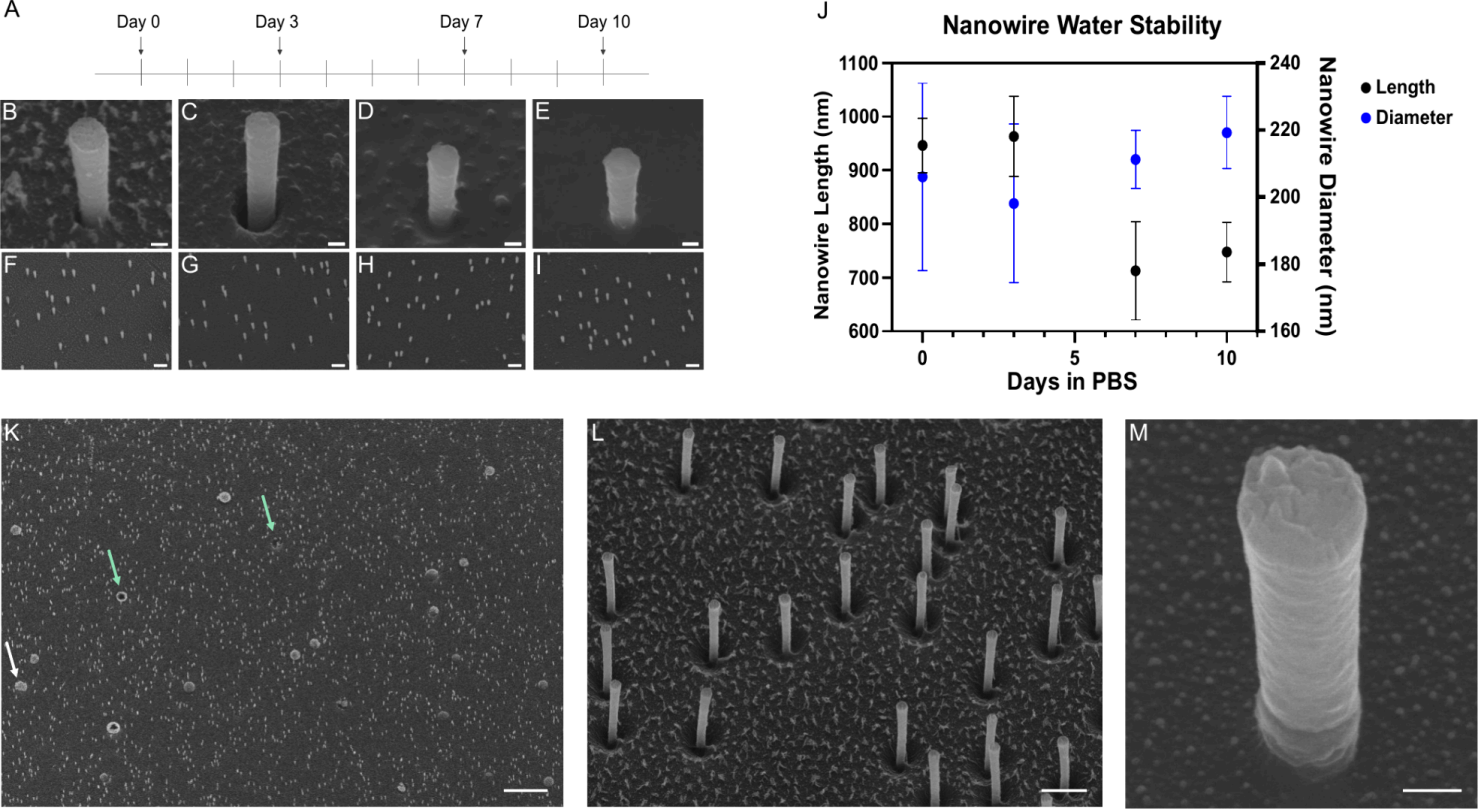
NW stability can be increased by incorporating trimers and electrofunctionalization or chemical strengthening. (A–J) NW stability after being submerged in PBS up to 10 days. (A) Timeline of PBS exposure to NWs. (B–E) 30° tilted SEM images of NW strengthened with trimers and 100 mM Fe^3+^. NWs were imaged before exposure to PBS (B, F), after 3 days of exposure (C, G), after 7 days of exposure (D, H), and after 10 days of exposure (E, I). Lower magnification images of NWs after exposure available in Figure S6. (J) Graph illustrating the NW diameter and NW length at various time points. Dots on the graph represent the mean, while error bars represent the standard deviation. (K–M) 30° tilted view SEM images of NWs electrofunctionalized with ETE-S. In K, the white arrow indicates the plasticizers inherent to the TE membrane, and the green arrows indicate the holes left from the plasticizers. The scale bars are 100 nm for panels B–E and M, 1 μm for panels F–I and L, and 10 μm for panel K.

While the addition of cations (such as Fe^3+^) or electrofunctionalization with trimers can be used to increase the stability, maximum stability was achieved by using a combination of both cations and trimer incorporation (schematic in Figure S5). Since the higher ion concentration increases the viscosity of the polymer solution, iron ions were added after the initial pore filling with PEDOT-S mixed with ETE-S. This allowed the diffusion of iron ions into the pores to simultaneously cross-link the polymer and aid in trimer functionalization. Images of such NWs at day 0 and at different time points after exposure to PBS are shown in Figure 2B–I. For the first 3 days, no decrease in the NW diameter or length was observed. After 7 days, the length decreased by 25% (946 nm day 0 vs 713 nm day 7, *p*-value <0.0001), while the diameter did not significantly decrease, Figure 2J. This change in height could be due to NW decomposition, but there is also the possibility that height is decreased due to the accumulation of salt from the PBS on the TE membrane. Summarizing, these experiments have shown that the PEDOT-S NWs can be made water stable by adding cations and trimers.

The NWs remain conductive after processing, as mapped by conductive atomic force microscopy (cAFM). The NWs studied were composed of 5 mg/mL PEDOT-S and 5 mM Fe^3+^ in PBS. In cAFM, a Pt/Ir coated tip is scanned across the sample, resulting in the simultaneous acquisition of topographic information and electrical current flow between the tip and the biased sample. Samples with either NWs lying flat on a surface (Figure 3A–D) or NWs extending from a PI membrane (Figure 3E–H) were mapped. For the NWs lying down, NWs were stochastically placed onto prepatterned Au lines on glass. The Au lines were contacted, allowing an electrical current to flow between the tip and the NWs. Distance dependent conductivity was found, with sections of the NW closer to the gold electrode showing a higher current, Figure 3D.

**Figure 3:**
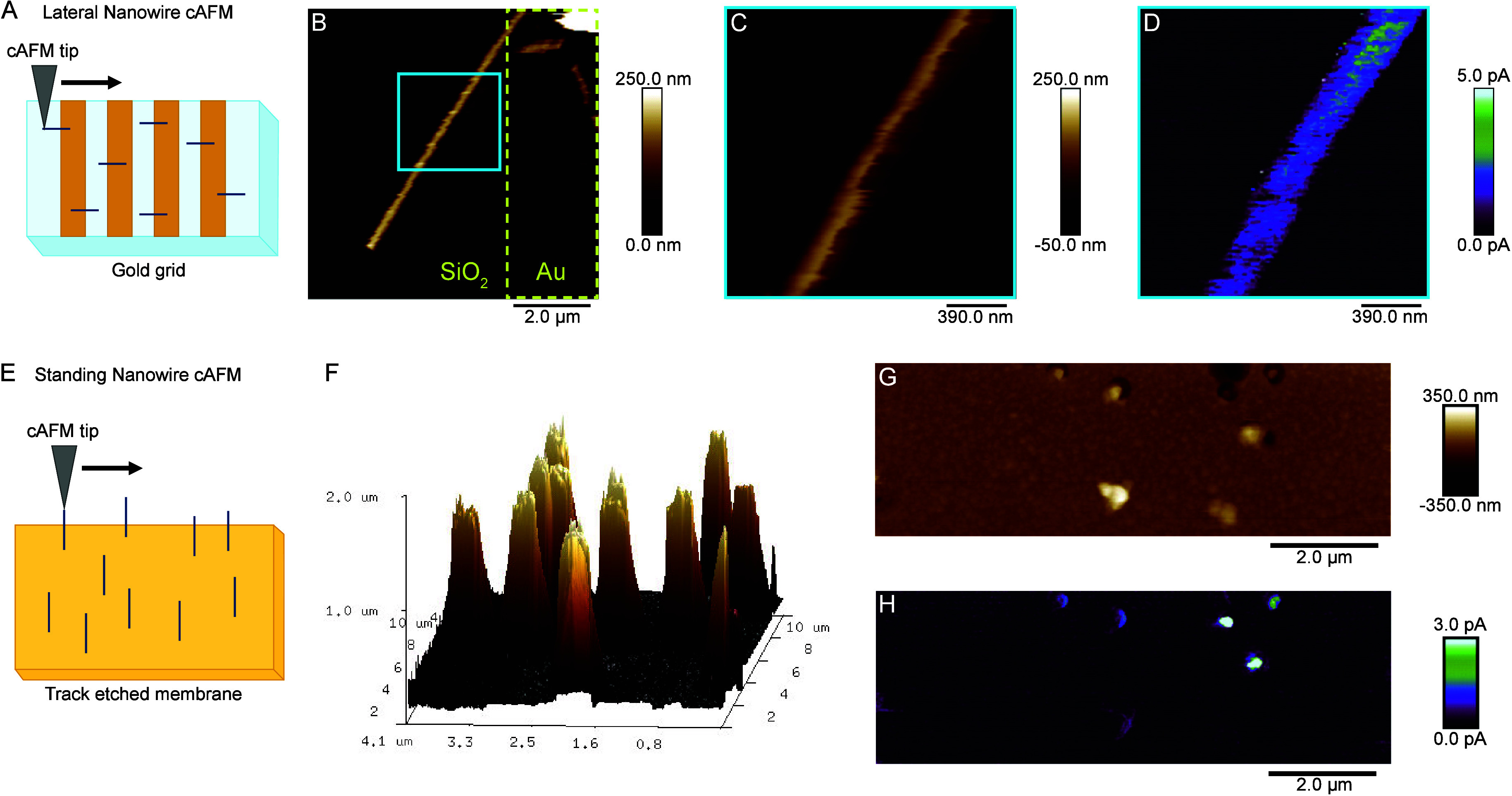
Physical characterization of NWs was performed by conductive atomic force microscopy (cAFM). Panels A and E are diagrams illustrating cAFM configuration, panels B–D are measurements taken on NWs lying flat on a surface, while panels F–H are measurements on standing NWs still embedded in a TE membrane. Topography images, both in 2D (B, C, G) and 3D (F) representation, range from white at the highest point, brown at midpoint, to black at the lowest point. Current maps (D, H) express light green at the most conductive point, then range through blue, and purple at the midpoints, then to black at the least conductive point. (B) Topography image of NW height. The image shows a single NW lying on a patterned surface, part of the NW on the gold section, and the rest of the NW on the glass surface. The gold section is denoted by the dotted yellow line, while the rest of the image is the glass surface. (C, D) Topography image of height (C) and current map (D) on a section of the same NW as shown in B. (F) Three-dimensional representation of cAFM height measurements on NWs. (G, H) Two-dimensional topography (G) and current map (H) on standing NWs.

For NWs standing up (Figure 3E), a thin layer of silver paint was used to contact the bottom of the membrane. The cAFM probe was then used to scan the top of the standing spikes. Scanning on standing wires poses a significant challenge for cAFM due to the high aspect ratio, which can lead to bending or breakage of the NWs when they contact the probe tip. For this reason, we used shorter NWs. Currents in the low pA regime were detected when scanning over the NWs, Figures 3D and S7. In comparison, no current over the noise level was registered when the tip was on the naked polymer membrane. The silver paint back contact opens up avenues to incorporate the membrane on pixel arrays for individually addressable nanowires, Figure S9.

In the lateral configuration, we observed current flow multiple μm away from the contact, Figure 3D. In the vertical configuration, the current flows from the tip through the NW inside the pores before reaching the back contact, thereby extending 12 μm in total. Importantly, this highlights that the polymer inside the pores is electrically continuous throughout its length. Compensating for the large distance, a higher voltage was used (10 V) in the vertical configuration, as opposed to the lateral one (5 V). Further optimization of contact chemistry and geometry will likely decrease the observed resistance since PEDOT-S in two-dimensional transistor setups has shown high conductivity.^26,17^ Taken together, the cAFM results show that the NWs are conductive and can be contacted on the backside of the membrane.

After establishing the possibility of generating water-stable and conductive NWs, we interfaced them with living cells. The NW membrane was fixed to the bottom of a biocompatible polycarbonate tube, forming an easy-to-handle cell culture vessel. Two vastly different cell types were explored in separate experiments: the single cell algal model system *Chlamydomonas reinhardtii* and clinically relevant human primary hematopoietic stem and progenitor cells (HSPCs, CD34^+^), Figures 4 and S8. To enable high-resolution SEM imaging of the NW–cell interface, samples were carefully fixed, dehydrated, and point-dried to retain their native morphology and minimize the risk of geometrical changes.

**Figure 4:**
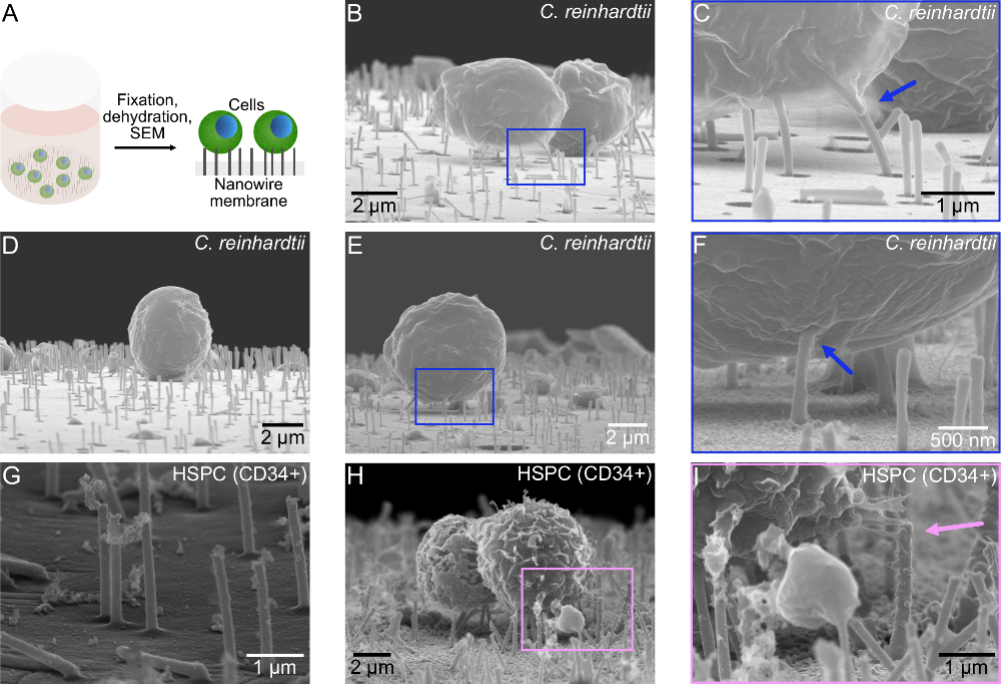
Interfacing living cells with CP NWs stabilized by 10 mM Fe^3+^. (A) Schematic depicting cells added to a culture vessel incorporating the NW membrane in the bottom. Following fixation and dehydration, the sample is imaged from the side to reveal the NWs interfacing with the cells. (B–F) SEM images showing algal cells, *C. reinhardtii*, on the NWs. Panels C, F, I are zoomed in from the marked regions in panels B, E, H. Blue arrows show NWs seemingly reaching inside the cells. (G–I) SEM images showing human primary hematopoietic stem cells (HSPCs, CD34^+^) interfacing NWs. Image G was obtained in a cell-free region. The pink arrow in I depicts cells reaching out to grab the spikes.

Interfacing nanowires with the sweet water algae *C. reinhardtii* poses a challenge for the NWs’ water stability since the cells’ preferred medium is hypo-osmolar and will more readily degrade the NWs. The cells were dispersed in dilute PBS (20%, in water), added to the culture wells, and centrifuged onto the NWs at 600 g to achieve a controlled pelleting without risk of cell aggregation. Despite the centrifugation induced pressure, SEM images revealed NWs extending vertically in areas with and without cells, Figure 4B–F. Some broken NWs were observed, which could be related to mechanical impact from the cells, fluid exchanges during fixation, or dehydration. There was no visible NW degradation due to hypo-osmolarity of the medium. Here, we note that cell medium will contain sugars and more (divalent) cations than PBS and will therefore further stabilize the nanowires. Interestingly, NWs close to the cells bent toward the cell membrane and were seemingly reaching intracellularly, Figure 4C,F. Nanostructures of similar dimensions can spontaneously pierce the cell membrane,^22^ and owing to the electrically conductive nature of the NWs, the intimate cell–NW contact can relay external voltage pulses for local destabilization of the cell membrane^27^ thereby enhancing the possibility for intracellular access.

Human primary HSPCs are defined by a surface marker, CD34^+^, and are routinely used in the clinic as bone marrow transplants. We have previously described how tubular nanostructures, nanostraws, could be used to access the intracellular space of HSPCs without being noticed by the cellular defense systems.^6^ Apart from this, little is known about how these cells interface with axially elongated nanostructures, despite their clinical relevance. Similar to the experiments with the algae, cells were centrifuged onto the NWs and subsequently prepared for SEM, Figure 4G–I. Importantly, these sensitive cells were dispersed in an isotonic medium. Even after rinsing, fixation, dehydration, and critical point drying, the NWs still protruded out from the NW membrane. Compared to the algal samples, more debris was observed, possibly being cell residues from the freeze–thaw cycle before being applied to the NWs, or coming from the cell medium. Cell morphology did not show any obvious signs of cell damage. It was repeatedly observed that cells close to NWs extended toward them, seemingly wanting to grab on, Figure 4I, as observed previously for other NW structures.^28,29^ In summary, the CP NWs can be made water stable and can be used to interact with living cells, both human and algal, and form close cell–NW interfaces.

In summary, we have demonstrated a lithography-free platform to generate PEDOT-S nanowires with controlled dimensions. By avoiding lithography, NWs can be produced at scale where the ICP reactor governs the sample size (typically a 4 in. or 6 in. sample size, depending on the reactor used). NWs were made water stable through the introduction of thiophene trimers and either cations or electrofunctionalization. When interfacing the cells with the NWs, they remained intact and could withstand 600 g of cell pelleting, even in hypo-osmolar solution.

The NW processing described here is not limited to use within cellular interfacing but is general in nature. The geometry and high surface-to-volume ratio could allow for making organic electrochemical transistors (OECTs) with very high switching speeds owing to an optimal gating geometry and short diffusion paths for ions moving in or out of the NWs. In addition, individually addressable nanowires could be achieved by using a pixel array as a back contact, Figure S9. These qualities open new doors for electrical and biological devices based on one-dimensional CPs.

## Data Availability

All data are available in the main text or the Supporting Information.

## Supporting Information Available

Methods section and additional figures describing processing, characterization, and systems implementation of the nanowires (PDF)

## Notes

Ethics Statement: Work with primary human CD34^+^ cells was approved by the regional ethical committee for Lund/Malmö (Regionala Etikprövningsnämnden i Lund/Malmö), approval no. 2010-696. Informed consent was obtained from mothers of umbilical cord blood donors, and all samples were deidentified prior to use in the study.

## References

[ref1] Zhang Z.; Lamers N.; Sun C.; Hetherington C.; Scheblykin I. G.; Wallentin J. Free-Standing Metal Halide Perovskite Nanowire Arrays with Blue-Green Heterostructures. Nano Lett. 2022, 22 (7), 2941–2947. 10.1021/acs.nanolett.2c00137. 35325539 PMC9011394

[ref2] Behrman K.; Kymissis I. Micro light-emitting diodes. Nature Electronics 2022, 5 (9), 564–573. 10.1038/s41928-022-00828-5.

[ref3] Ram M. S.; Persson K.-M.; Irish A.; Jönsson A.; Timm R.; Wernersson L.-E. High-density logic-in-memory devices using vertical indium arsenide nanowires on silicon. Nature Electronics 2021, 4 (12), 914–920. 10.1038/s41928-021-00688-5.

[ref4] Li Z.; Song J.; Mantini G.; Lu M.-Y.; Fang H.; Falconi C.; Chen L.-J.; Wang Z. L. Quantifying the Traction Force of a Single Cell by Aligned Silicon Nanowire Array. Nano Lett. 2009, 9 (10), 3575–3580. 10.1021/nl901774m. 19824706

[ref5] Cao Y.; Hjort M.; Chen H.; Birey F.; Leal-Ortiz S. A.; Han C. M.; Santiago J. G.; Paşca S. P.; Wu J. C.; Melosh N. A. Nondestructive nanostraw intracellular sampling for longitudinal cell monitoring. Proc. Natl. Acad. Sci. U. S. A. 2017, 114 (10), E1866–E1874. 10.1073/pnas.1615375114. 28223521 PMC5347600

[ref6] Schmiderer L.; Subramaniam A.; Žemaitis K.; Bäckström A.; Yudovich D.; Soboleva S.; Galeev R.; Prinz C. N.; Larsson J.; Hjort M. Efficient and nontoxic biomolecule delivery to primary human hematopoietic stem cells using nanostraws. Proc. Natl. Acad. Sci. U. S. A. 2020, 117 (35), 21267–21273. 10.1073/pnas.2001367117. 32817519 PMC7474688

[ref7] Andrei V.; Roh I.; Yang P. Nanowire photochemical diodes for artificial photosynthesis. Science Advances 2023, 9 (6), eade9044 10.1126/sciadv.ade9044. 36763656 PMC9917021

[ref8] Woo R. L.; Xiao R.; Kobayashi Y.; Gao L.; Goel N.; Hudait M. K.; Mallouk T. E.; Hicks R. F. Effect of Twinning on the Photoluminescence and Photoelectrochemical Properties of Indium Phosphide Nanowires Grown on Silicon (111). Nano Lett. 2008, 8 (12), 4664–4669. 10.1021/nl802433u. 19367937

[ref9] Kim M.; Kwon J.; Lee H. J.; Park K. S.; Kim J.; Kim J.; Baek K.; Yuan H.; Hyun J. K.; Cho Y. S.; et al. Bespoke selenium nanowires with comprehensive piezo-phototronic effects as viable p-type semiconductor-based piezo-photocatalysts. Nano Energy 2023, 114, 108680 10.1016/j.nanoen.2023.108680.

[ref10] Rathmell A. R.; Bergin S. M.; Hua Y. L.; Li Z. Y.; Wiley B. J. The Growth Mechanism of Copper Nanowires and Their Properties in Flexible, Transparent Conducting Films. Adv. Mater. 2010, 22 (32), 3558–3563. 10.1002/adma.201000775. 20512817

[ref11] Rathmell A. R.; Wiley B. J. The Synthesis and Coating of Long, Thin Copper Nanowires to Make Flexible, Transparent Conducting Films on Plastic Substrates. Adv. Mater. 2011, 23 (41), 4798–4803. 10.1002/adma.201102284. 21953576

[ref12] Liu H.; He Y.; Jin B.; Lang X.; Xie H.; Jiang Q. A compact lithiophilic dual metal oxide nanowire array on 3D copper mesh enables dendrite-free long-life lithium metal anodes. Chemical Engineering Journal 2024, 496, 154072 10.1016/j.cej.2024.154072.

[ref13] Chen H.; Yan R.; Chen Y.; Li S.; Sun T.; Zhou J.; Qian M.; Wang Z.; Lü Z. A solution-based oxidation–reduction approach for spontaneous construction of nanowire architectures on copper metals. Surfaces and Interfaces 2024, 46, 104125 10.1016/j.surfin.2024.104125.

[ref14] Yang S. Y.; Kim B. N.; Zakhidov A. A.; Taylor P. G.; Lee J. K.; Ober C. K.; Lindau M.; Malliaras G. G. Detection of Transmitter Release from Single Living Cells Using Conducting Polymer Microelectrodes. Adv. Mater. 2011, 23 (24), H184–H188. 10.1002/adma.201100035. 21400618 PMC3282049

[ref15] Del Agua I.; Marina S.; Pitsalidis C.; Mantione D.; Ferro M.; Iandolo D.; Sanchez-Sanchez A.; Malliaras G. G.; Owens R. M.; Mecerreyes D. Conducting Polymer Scaffolds Based on Poly(3,4-ethylenedioxythiophene) and Xanthan Gum for Live-Cell Monitoring. ACS Omega 2018, 3 (7), 7424–7431. 10.1021/acsomega.8b00458. 30087913 PMC6068595

[ref16] Chang J. F.; Gwinner M. C.; Caironi M.; Sakanoue T.; Sirringhaus H. Conjugated-Polymer-Based Lateral Heterostructures Defined by High-Resolution Photolithography. Adv. Funct. Mater. 2010, 20 (17), 2825–2832. 10.1002/adfm.201000436.

[ref17] Mousa A. H.; Bliman D.; Hiram Betancourt L.; Hellman K.; Ekström P.; Savvakis M.; Strakosas X.; Marko-Varga G.; Berggren M.; Hjort M.; et al. Method Matters: Exploring Alkoxysulfonate-Functionalized Poly(3,4-ethylenedioxythiophene) and Its Unintentional Self-Aggregating Copolymer toward Injectable Bioelectronics. Chem. Mater. 2022, 34 (6), 2752–2763. 10.1021/acs.chemmater.1c04342. 35360437 PMC8944941

[ref18] Hjort M.; Mousa A. H.; Bliman D.; Shameem M. A.; Hellman K.; Yadav A. S.; Ekström P.; Ek F.; Olsson R. In situ assembly of bioresorbable organic bioelectronics in the brain. Nat. Commun. 2023, 14 (1), 4453 10.1038/s41467-023-40175-3. 37488105 PMC10366153

[ref19] Xie X.; Xu A. M.; Angle M. R.; Tayebi N.; Verma P.; Melosh N. A. Mechanical Model of Vertical Nanowire Cell Penetration. Nano Lett. 2013, 13 (12), 6002–6008. 10.1021/nl403201a. 24237230

[ref20] Liu R.; Lee J.; Tchoe Y.; Pre D.; Bourhis A. M.; D’Antonio-Chronowska A.; Robin G.; Lee S. H.; Ro Y. G.; Vatsyayan R.; et al. Ultra-Sharp Nanowire Arrays Natively Permeate, Record, and Stimulate Intracellular Activity in Neuronal and Cardiac Networks. Adv. Funct. Mater. 2022, 32 (8), 2108378 10.1002/adfm.202108378. 35603230 PMC9122115

[ref21] Persson H.; Li Z.; Tegenfeldt J. O.; Oredsson S.; Prinz C. N. From immobilized cells to motile cells on a bed-of-nails: effects of vertical nanowire array density on cell behaviour. Sci. Rep. 2015, 5 (1), 18535 10.1038/srep18535. 26691936 PMC4686997

[ref22] Xu A. M.; Aalipour A.; Leal-Ortiz S.; Mekhdjian A. H.; Xie X.; Dunn A. R.; Garner C. C.; Melosh N. A. Quantification of nanowire penetration into living cells. Nat. Commun. 2014, 5 (1), 3613 10.1038/ncomms4613. 24710350 PMC6057472

[ref23] Aydemir U.; Mousa A. H.; Dicko C.; Strakosas X.; Shameem M. A.; Hellman K.; Yadav A. S.; Ekström P.; Hughes D.; Ek F. In situ assembly of an injectable cardiac stimulator. Nat. Commun. 2024, 15 (1), 6774 10.1038/s41467-024-51111-4. 39117721 PMC11310494

[ref24] Gerasimov J. Y.; Halder A.; Mousa A. H.; Ghosh S.; Harikesh P. C.; Abrahamsson T.; Bliman D.; Strandberg J.; Massetti M.; Zozoulenko I.; et al. Rational Materials Design for In Operando Electropolymerization of Evolvable Organic Electrochemical Transistors. Adv. Funct. Mater. 2022, 32 (32), 2202292 10.1002/adfm.202202292.

[ref25] Strakosas X.; Biesmans H.; Abrahamsson T.; Hellman K.; Ejneby M. S.; Donahue M. J.; Ekström P.; Ek F.; Savvakis M.; Hjort M.; et al. Metabolite-induced in vivo fabrication of substrate-free organic bioelectronics. Science 2023, 379 (6634), 795–802. 10.1126/science.adc9998. 36821679

[ref26] Yano H.; Kudo K.; Marumo K.; Okuzaki H. Fully soluble self-doped poly(3,4-ethylenedioxythiophene) with an electrical conductivity greater than 1000 S cm^–1^. Science Advances 2019, 5 (4), eaav9492 10.1126/sciadv.aav9492. 30993206 PMC6461456

[ref27] Xie C.; Lin Z.; Hanson L.; Cui Y.; Cui B. Intracellular recording of action potentials by nanopillar electroporation. Nat. Nanotechnol. 2012, 7 (3), 185–190. 10.1038/nnano.2012.8. 22327876 PMC3356686

[ref28] Paulitschke P.; Keber F.; Lebedev A.; Stephan J.; Lorenz H.; Hasselmann S.; Heinrich D.; Weig E. M. Ultraflexible Nanowire Array for Label- and Distortion-Free Cellular Force Tracking. Nano Lett. 2019, 19 (4), 2207–2214. 10.1021/acs.nanolett.8b02568. 30427688

[ref29] Xie X.; Aalipour A.; Gupta S. V.; Melosh N. A. Determining the Time Window for Dynamic Nanowire Cell Penetration Processes. ACS Nano 2015, 9 (12), 11667–11677. 10.1021/acsnano.5b05498. 26554425

