# Supplementary material for: Lithography-Free Water Stable Conductive Polymer Nanowires
Source: Nano Lett. 2025 Feb 13;25(8):3059–65. doi: 10.1021/acs.nanolett.4c05016 (PMC11869357; doi:10.1021/acs.nanolett.4c05016)
Supplement: Supplementary file 1 — nl4c05016_si_001.pdf [file nl4c05016_si_001.pdf]

Supplementary Materials for

Lithography-free Water Stable Conductive Polymer Nanowires

Authors: Damien Hughes[1], Abdelrazek H. Mousa[2&], Chiara Musumeci[3], Malte Larsson[1], Muhammad Anwar Shameem[2], Umut Aydemir[1], Ludwig Schmiderer[4§], Jonas Larsson[4], Magnus Berggren[3], Fredrik Ek[1,5], Roger Olsson[1,2,5], and Martin Hjort[1,5,*]

Affiliations:

[1] Chemical Biology & Therapeutics, Department of Experimental Medical Science, Lund University, SE-221 84, Lund, Sweden

[2] Department of Chemistry and Molecular Biology, University of Gothenburg, SE-405 30, Gothenburg, Sweden

[3] Laboratory of Organic Electronics, Department of Science and Technology, Linköping University, SE-60174, Norrkoping, Sweden

[4] Division of Molecular Medicine and Gene Therapy, Department of Laboratory Medicine and Lund Stem Cell Center, Lund University, SE-221 00, Lund, Sweden

[5] Chemical Biology Consortium Sweden (CBCS), Karolinska Institute, S-171 21, Stockholm, Sweden

[&] Current affiliation: Chemistry Department, King Fahd University of Petroleum and Minerals, Dhahran 31261, Saudi Arabia

[§] Current affiliation: Institute for Stem Cell Biology and Regenerative Medicine, Stanford University, Stanford, CA, USA

*Corresponding author. Email: martin.hjort@med.lu.se

Methods

NW formation: A commercially available poly(3,4-ethylenedioxythiophene)butoxy-1-sulfonate (PEDOT-S) solution (Clevios KS LVW 2012, Hereus Epurio Innovation) was applied onto track-etched (TE) polyimide (PI) and polycarbonate (PC) membranes. TE membranes with different pore geometries were evaluated: diameters ranging from 160–200 nm, pore densities 2–5.5 e^7 cm^{-2}, and thickness of 12–25 um (It4IP S.A., Louvain-la-Neuve, Belgium).

In some experiments, different salts were added to the polymer to increase the ionic strength of the solution. To ensure a well-dispersed solution, the PEDOT-S was ultrasonicated before adding it to the TE membrane in order to cover both the top of the membrane and to fill up the nanopores. The polymer solution was left to dry overnight followed by mechanical removal of excess (dry) polymer residing on top of the TE membrane using masking tape (Heavy Duty Masking tape, 3M). The polymer filled TE membrane was electrostatically or mechanically adhered to a 4'' Si wafer using an electrostatic gun (Sigma-Aldrich, Zerostat anti-static instrument) or Kapton tape for subsequent semiconductor processing.

ICP-RIE etching: Oxygen based inductively couple plasma reactive ion etching (ICP-RIE) was used to selectively remove TE membrane in an anisotropic manner (Plasma-Therm, APEX SLR). Processing parameters for standard spikes using ICP-RIE: 50 sccm O_2, 25 W RF, 500 W ICP, 10 mTorr reactor pressure, 120-180 s etch duration. Processing parameters for a 'strong' etch using ICP-RIE: 50 sccm O_2, 50 W RF, 600 W ICP, 10 mTorr reactor pressure, 180 s etch duration. The duration of this etching step governs the length of the finished NW and was varied to extract an etch rate.

Polymer solution for electrofunctionalization and chemical functionalization: Conductive polymer solution was prepared by dissolving 10 mg/ml 4-(2-(2,5-bis(2,3-dihydrothieno[3,4-b][1,4]dioxin-5-yl)thiophene-3-yl)ethoxy)butane-1-sulfonate (ETE-S), 5 mg/ml PEDOT-S (PEDOT-S; Sample: Clevios KS LVW 2012, Hereus Epurio Innovation), and iron (III) sulfate hydrate (Sigma-Aldrich; Final concentration in solution 5 mM) in phosphate buffered saline (PBS). For chemical functionalization, CP solution only contained 10 mg/ml ETE-S, 5 mg/mL PEDOT-S in PBS. To ensure a well-dispersed and low viscosity solution, samples were ultrasonicated 5 times (20 pulses at 100 % amplitude and 0.75 cycle; Hielscher).

Electrofunctionalization: Electrofunctionalization was performed by pipetting CP solution onto a polyimide membrane which was adhered to a piece of conductive copper tape. A Keithley Sourcemeter (Model 2612B) was used to apply a 0.9 V electrical bias in between the CP solution (AgCl dip-in electrode) and the copper tape for 30–60 minutes. The resulting current was monitored throughout the electrofunctionalization process. After electrofunctionalization sample was allowed to air dry overnight.

To remove the copper tape, the membrane was submerged in 99.5 % ethanol (Solveco). Possible adhesive residues were removed from the back of the membrane by using a Kimwipe (Kimtech) and 99.5 % ethanol. Excess alcohol was left to evaporate. Excess CP was mechanically delaminated using tape (Heavy Duty Masking tape, 3M). The top surface was cleaned using a Kimwipe with 99.5 % ethanol to remove possible contaminants. Following this, the electrofunctionalized samples were processed using standard ICP-RIE as described before.

Chemical functionalization: CP solution was pipetted onto a PI membrane and allowed to dry for an hour, before the addition of 100 mM iron (III) sulfate hydrate (Sigma-Aldrich). Sample was allowed to dry and further processed as a standard sample.

SEM Imaging: For scanning electron microscopy (SEM), small pieces from the NW membrane were cut out and sputter coated (Q150T ES, Quorum) with 5 nm of Pt:Pd (80:20) alloy. Samples were imaged using cold-field emission SEM (SU8010, Hitachi) operating at 10 kV. NW diameter and NW length were measured in ImageJ software (Version 1.54g) using a 30° tilted view 10,000x magnification SEM image of each sample. Measured length was multiplied by a factor of two, to accommodate for tilt. N for each sample point varies between 14 and 33, all NWs completely visible, intact, and in frame of the image were measured.

For SEM of cells and algae on NWs, additional sample preparation was needed.
Human primary stem and progenitor cells (CD34+) cells were dispersed in PBS or algal cells from the strain *Chlamydomonas Reinhardtii* dispersed in dilute PBS (0.2x) were centrifuged onto the NW membrane followed by fixation in 2 % glutaraldehyde, 2 % formaldehyde in PBS. After

fixation, cells were stored in Sorensen's phosphate buffer. An ethanol exchange series of 10 min each in 30 %, 50 %, 75 %, 90 %, 95 %, and 99.5 % ethanol was used. Critical point drying (CPD) was used to completely remove the ethanol from the cells with minimal disruption of the cellular shape in a Quorum K850 CPD (Quorum Technologies). The CP dried samples were coated with 5 nm Pt:Pd and imaged using SEM.

NW stability in phosphate buffered saline: Samples were taped into a Petri dish (Avantor), which was then filled with 3 ml of PBS. This formed a layer of PBS over the samples. PBS was removed from Petri dish, by gently tilting and pouring out the PBS. In order to remove residual PBS, 5 rinses of 1 ml milli-Q water was done, then 3 rinses of 1 ml of 99.7 % ethanol was done. Excess liquid from each rinse was removed by tilting the Petri dish, and using a Kimwipe to absorb liquid at the edge of the dish if the solution does not readily evaporate. Ethanol was allowed to evaporate after the last rinse. Visualization of NWs was done via SEM as described in the 'SEM imaging' section.

Conductive Atomic Force Microscopy

C-AFM was performed on a Dimension Icon XR equipped with a PF-TUNA module (current sensitivity 20 pA/V) from Bruker. Pt/Ir-coated silicon probes (k = 3 N/m) were used to simultaneously map topography and current in PF-TUNA mode. The current maps were obtained by constantly biasing the Au substrate, while keeping the scanning AFM probe at ground. All the measurements were performed at room temperature in ambient atmosphere.

Cell culture:

Stem and progenitor cells: Umbilical cord blood was collected at Skåne University Hospital and Helsingborg Hospital. Mononuclear cells were extracted using Lymphoprep tubes (Alere Technologies, no. 1019818). CD34+ cells from mixed donors were isolated from mononuclear cells with a CD34 MicroBead Kit (Miltenyi Biotec, no. 130–046-703) according to the manufacturer's instructions. Cells were cells were frozen in 90 % fetal calf serum + 10 % dimethyl sulfoxide before use, and then thawed for the experiment. After thawing, cells were cultured using StemSpan serum-free expansion medium (Stemcell Technologies, no. 09650) with human stem cell factor (PeproTech, no. 300–07), human thrombopoietin (PeproTech, no. 300–18), and human recombinant Human Flt3-Ligand (PeproTech, no. 300–19), 100 ng/ml, 37 °C,

5% CO_2. Cells were washed in PBS and centrifuged at 150 g for 10 min to remove debris. The cell pellet was resuspended in PBS and centrifuged onto NWs at 600 g for 3 min.

Algal Cells: A cell suspension of the model cell line *Chlamydomonas reinhardtii* was suspended in 20 % PBS and centrifuged at 350 g for 10 min to remove debris. The cell pellet was resuspended in 20 % PBS and centrifuged onto NWs at 600 g for 3 min.

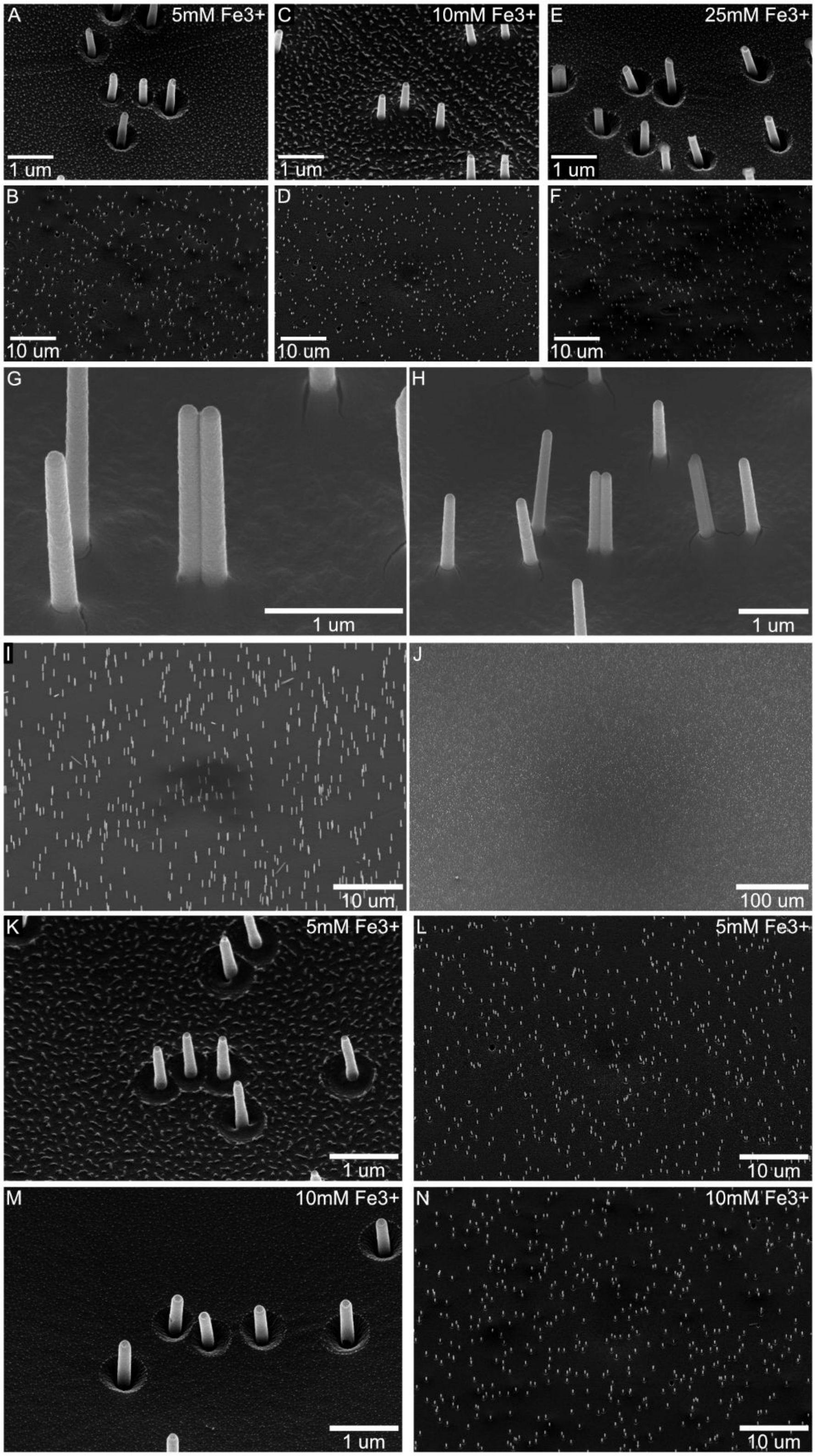

Figure S1: 30° tilted view SEM images depicting nanowires made with varying amounts of Fe^{3+} added during templating. PEDOT-S concentration used was 5 mg/ml for A–J, and 10 mg/ml for

K–N. A, B are made with 5 mM Fe^{3+}; C, D are made with 10 mM Fe^{3+}; E, F are made with 25 mM Fe^{3+}. Panels G–J include 30° tilted view SEM images depicting nanowires going from high to low magnification. Please note the excellent uniformity over large areas. Nanowires are made with 5 mg/ml PEDOT-S and 20 mM Fe^{3+}. Panels K–N include 30° tilted view SEM images with nanowires made with 10 mg/ml PEDOT-S with 5 mM Fe^{3+} (K, L), and with 10 mg/ml PEDOT-S with 10 mM Fe^{3+} (M, N).

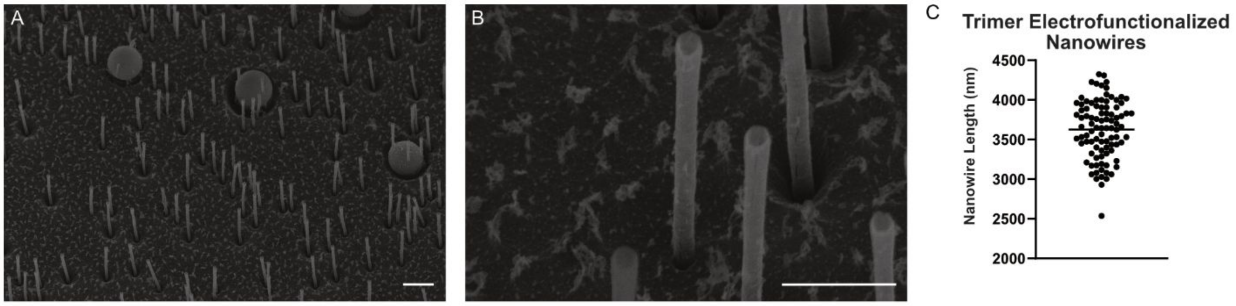

Figure S2: 30° tilted view SEM images of nanowires grown up to 4 μm using trimer incorporation and electrofunctionalization. Nanowires did not undergo ethanol rinse after delamination. (A) Scale bar 2 μm in panel A, and 1 μm in panel B. (C) Graph of observed lengths of nanowires in panel A. Variation in nanowire observed length is likely due to deviations in viewing angle. Each dot represents one nanowire, n = 93. Line illustrates mean.

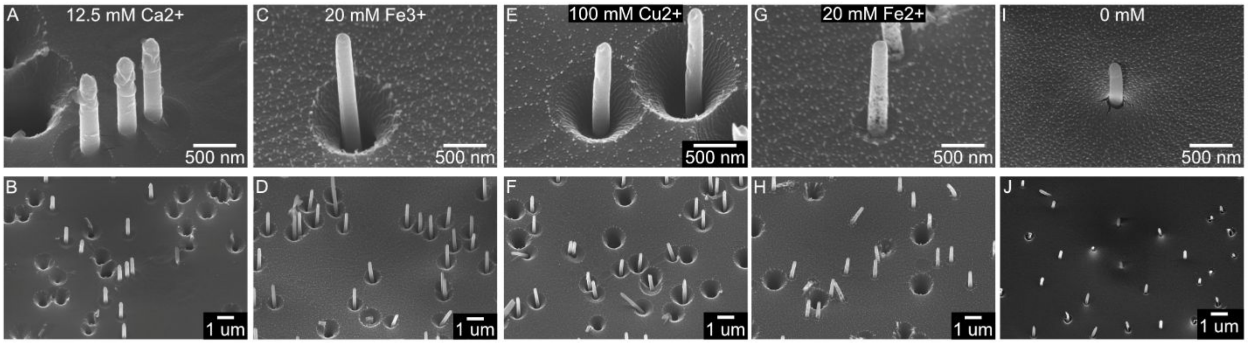

Figure S3. 30° tilted view SEM images depicting nanowires made with different ions added during templating (non-optimized settings). Nanowires in panels A, B are made with 12.5 mM Ca^{2+}; C, D are made with 20 mM Fe^{3+}; E, F are made with 100 mM Cu^{2+}; G, H are made with 20 mM Fe^{2+}; I, J are made without any additions.

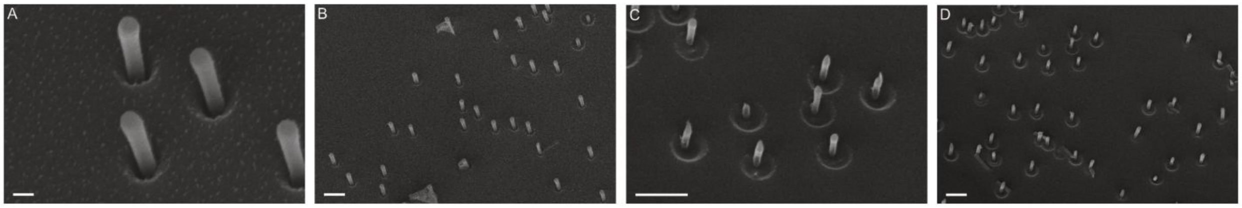

Figure S4: 30° tilted view SEM images illustrating PBS stability of nanowires composed of PEDOT-S with low levels of iron. Nanowires were composed of 5 mg/ml PEDOT-S and 5 mM Fe^{3+}. SEM images depicting nanowires before PBS exposure (A–B) and exposed to PBS for 24 hours (C–D). In panels C, D, degradation of nanowire tips is visible, as well as multiple displaced nanowires. For panel A Scale bar is 200 nm, for panels B–D scale bars are 1 μm.

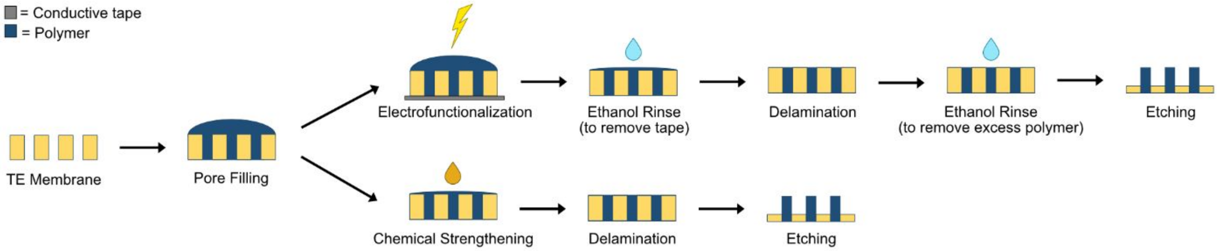

Figure S5: Schematic side-view describing incorporation thiophene trimers into nanowires and subsequent processing. A track-etched (TE) membrane is filled with conductive polymer solution. The sample then either undergoes electrofunctionalization or chemical strengthening. For electrofunctionalization, a 0.9 V bias is applied via piece of conductive tape, with a AgCl dip-in electrode as the counter electrode for 30-60 min. Sample is then allowed to dry overnight. An ethanol rinse is used to remove the sample from the conductive tape, and remove residual adhesive. After ethanol has evaporated, the sample is then delaminated to remove the top layer of polymer. Sample is then rinsed again with ethanol, to remove residual polymer. The TE membrane is then etched by oxygen via inductively coupled plasma reaction ion etching (ICP-RIE) to reveal the nanowires. For chemical strengthening, a solution of 100 mM Fe^{3+} is added to the surface of the sample. Sample is then allowed to dry overnight. Sample is then delaminated, then etched via standard ICP-RIE etching. Please note that schematic is not to scale.

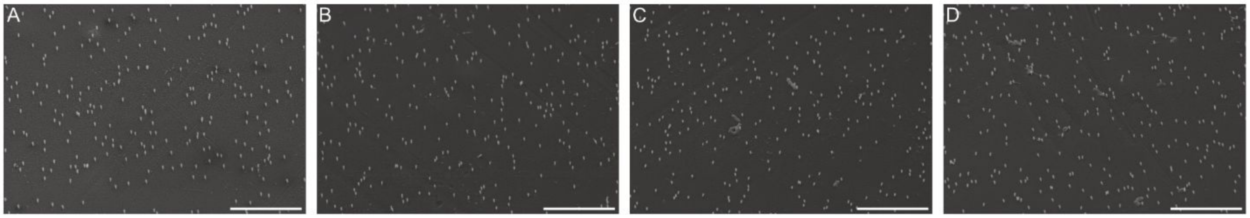

Figure S6: 30° tilted view SEM images of nanowire stability after exposure to PBS. Nanowires are stable in PBS up to 10 days. SEM images are from the same respective samples as Figure 2B–I, only imaged at a lower magnification. (A–D) Nanowires were imaged before exposure to PBS (Day 0, A), after three days of exposure (Day 3, B), after 7 days of exposure (Day 7, C) and after 10 days of exposure (Day 10, D). Scale bars are 10 microns.

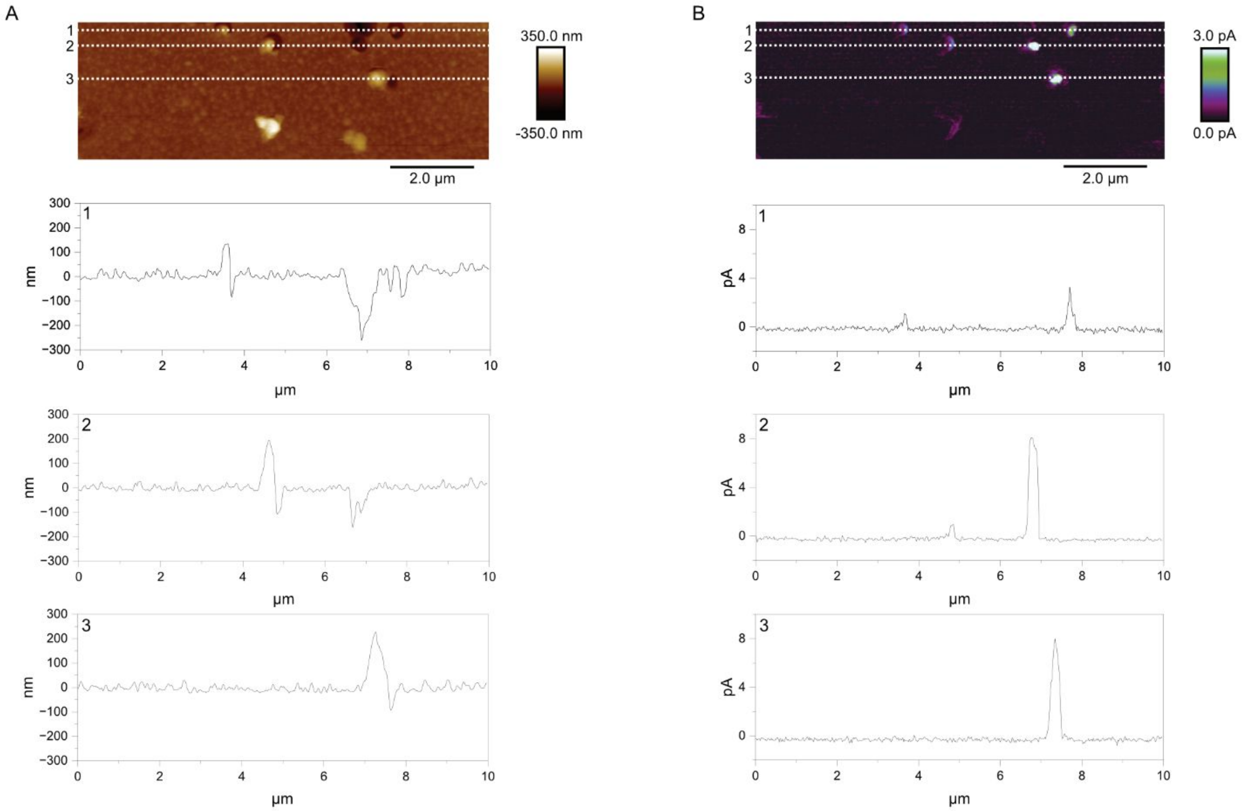

Figure S7: CAFM data for standing nanowires. Panel A is a topography image illustrating height and panel B is current map demonstrating current (B) together with section profiles along the lines marked in the maps (1-3).

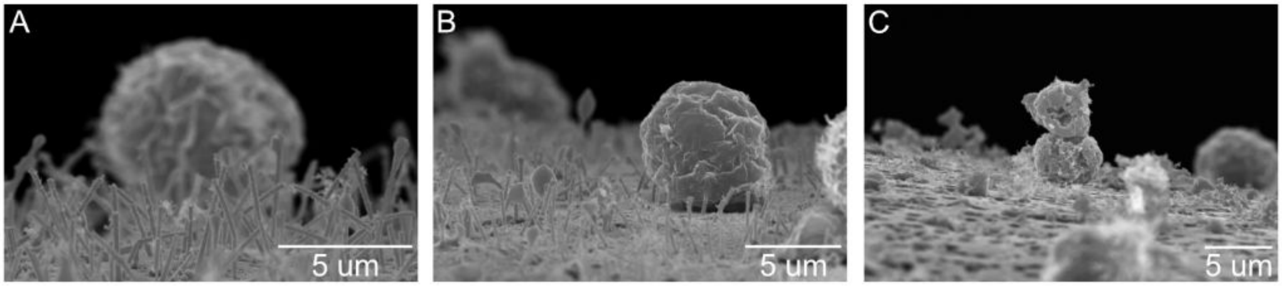

Figure S8: SEM images depicting CD34+ cells on nanowires (stabilized with 10mM Fe^{3+}). A, B depicts cells after successful dehydration and critical point drying whereas C shows cells after unsuccessful SEM preparation/dehydration.

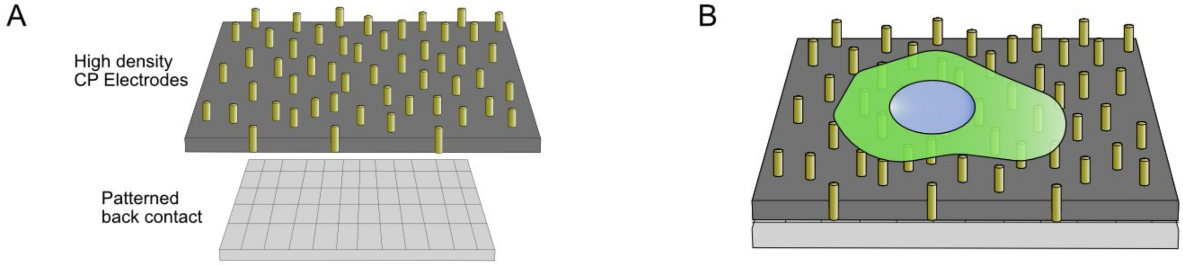

Figure S9: Diagram illustrating a method to achieve spatially addressable nanowires by adhering the nanowire membrane onto a patterned back contact, e.g. a CMOS pixel array. This enables a stochastic number of nanowires to be contacted by each pixel. The density of the initial template membrane governs the density of the nanowires and therefore the number of nanowires per pixel. A global top contact can be placed inside the cell medium to complete the circuit.

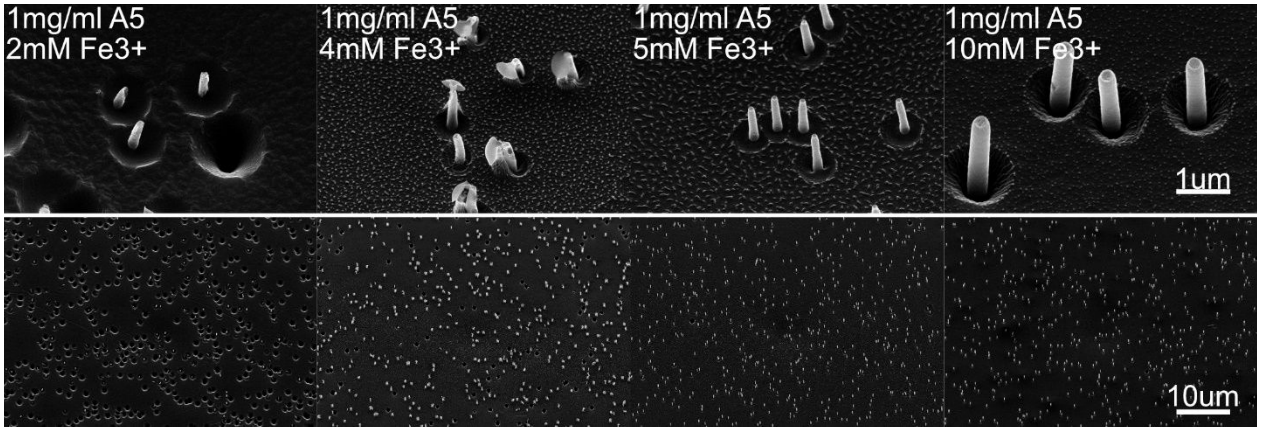

Figure S10: 30degree tilted view SEM images of nanowires made with low PEDOT-S concentration (1 mg/ml) and varied Fe^{3+} concentration (2 mM to 10 mM) as indicated in the figure. Top images show high magnification, bottom images show low magnification.

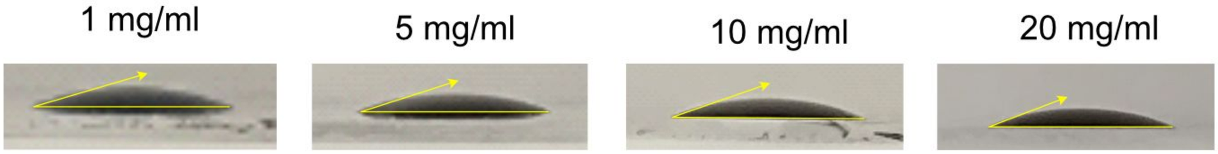

Figure S11: Contact angle measurements on PEDOT-S droplets on a glass slide. PEDOT-S concentration as indicated in the figure, diluted in water. Contact angle was found to be around 20° for all samples.
